# Syntheses and structures of two anthracene–benzoic acid derivatives as potential MOF linkers

**DOI:** 10.1107/S2056989025008503

**Published:** 2025-10-28

**Authors:** Shagun Kushwaha, Amit Thapliyal, Anil K. Mishra, Udai P. Singh, Ray J. Butcher

**Affiliations:** a Department of Chemistry Indian Institute of Technology Roorkee,Roorkee - 247667 India; b Department of Chemistry Howard University, 525 College St NW Washington DC 20059 USA; University of Aberdeen, United Kingdom

**Keywords:** crystal structure, anthracene derivatized ligands

## Abstract

In the title compounds, 2,2′-{[anthracene-9,10-diylbis(methyl­ene)]bis­(sulfane­di­yl)}di­benzoic acid di­methyl­acetamide tetra­solvate, C_30_H_22_O_4_S_2_·4C_4_H_9_NO **1** and 4,4′-{[anthracene-9,10-diylbis(methyl­ene)]bis­(­oxy)}di­benzoic acid di­methyl­formamide disolvate, C_30_H_22_O_6_·2C_3_H_7_NO **2**, the complete anthracene–benzoic acid mol­ecule is generated by a crystallographic centre of symmetry. The dihedral angle between the anthracene ring system and the pendant ring is 71.43 (7) in **1** and 75.27 (12)° in **2**. In the extended structures of **1** and **2**, O—H⋯O hydrogen bonds link the main mol­ecules into pairs of solvent mol­ecules to generate trimers. Weak C—H⋯O and C—H⋯π inter­actions further consolidate both structures.

## Abstract 2

In the title compounds, 2,2′-{[anthracene-9,10-diylbis(methyl­ene)]bis­(sulfanedi­yl)}di­benzoic acid di­methyl­acetamide tetra­solvate, C_30_H_22_O_4_S_2_·4C_4_H_9_NO **1** and 4,4′-{[anthracene-9,10-diylbis(methyl­ene)]bis­(­oxy)}di­benzoic acid di­methyl­formamide disolvate, C_30_H_22_O_6_·2C_3_H_7_NO **2**, the complete anthracene–benzoic acid mol­ecule is generated by a crystallographic centre of symmetry. The dihedral angle between the anthracene ring system and the pendant ring is 71.43 (7) in **1** and 75.27 (12)° in **2**. In the extended structures of both compounds, O—H⋯O hydrogen bonds link the main mol­ecules into pairs of solvent mol­ecules to generate trimers.

## 1. Chemical context

Anthracene is a rigid and planar tricyclic aromatic hydro­carbon, has extensive π-conjugation, high photoluminescence efficiency and good thermal stability (Klosterman *et al.*, 2009 ▸; Hunter *et al.*, 2001 ▸). The anthracene core serves as a light-harvesting chromophore and a versatile structural unit for designing functional materials such as organic semiconductors, photoresponsive switches, fluorescent sensors and supra­molecular hosts (Desiraju & Gavezzotti, 1989 ▸). In coordination chemistry, the incorporation of anthracene into ligands can significantly influence both the structural and electronic characteristics of the resulting complexes as reported by our group (*e.g.*, Verma *et al.*, 2022 ▸) and others (*e.g.*, Jindal *et al.*, 2021 ▸).
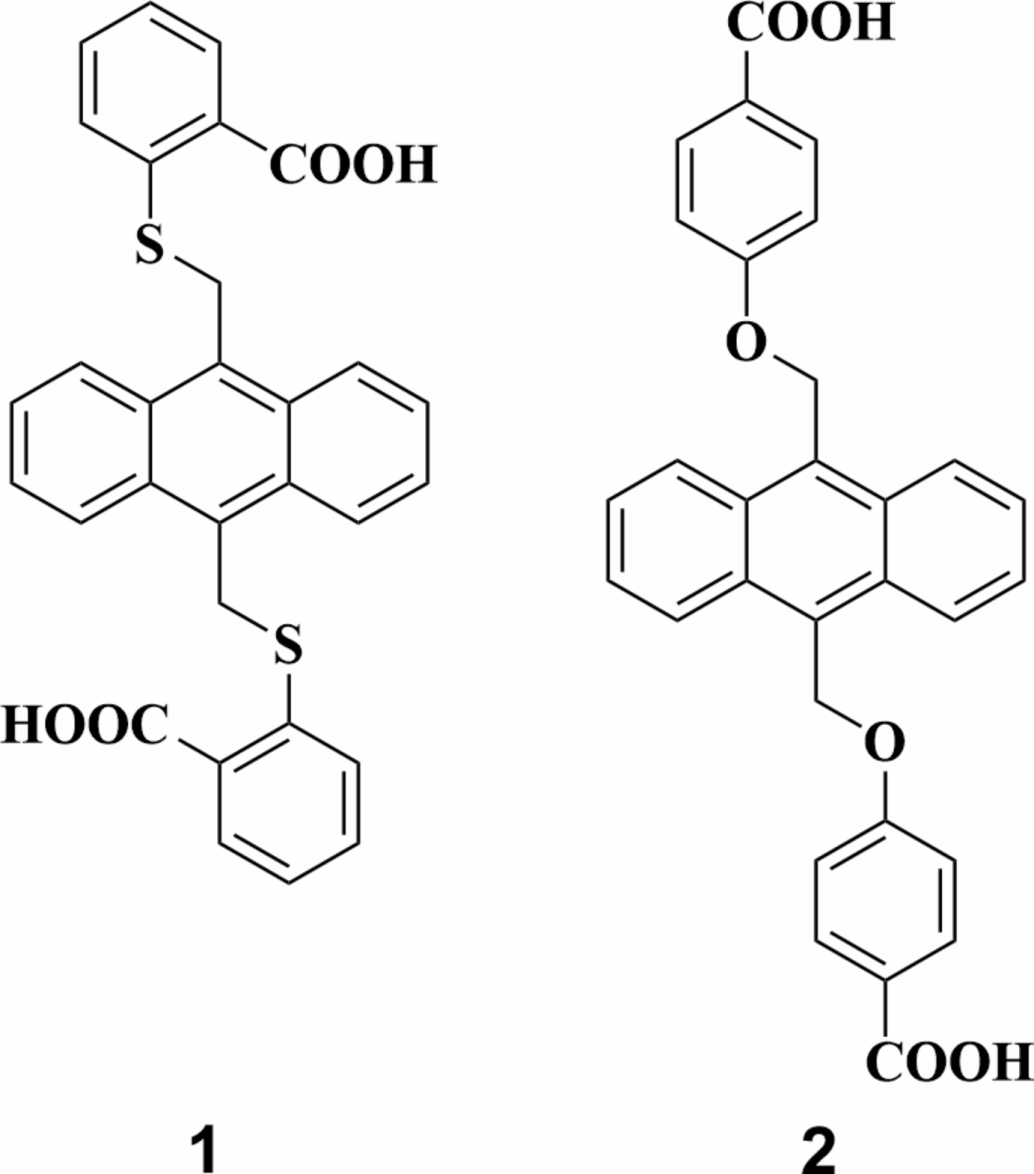


Herein we report the syntheses and crystal structures of the title compounds, **1** and **2**, with a thio­ether and ether linkage between the anthracene ring system and benzoic acid moiety, respectively. These compounds could act as potential linkers in metal–organic frameworks (MOFs) after deprotonation of the carb­oxy­lic acid.

## 2. Structural commentary

**Figure 1:**
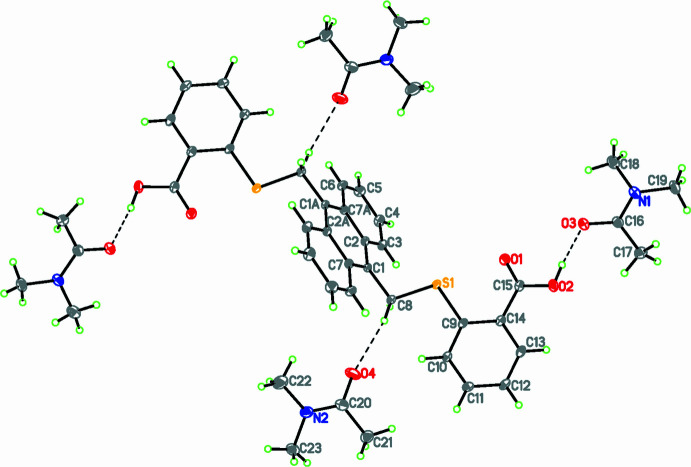
The mol­ecular structure of **1** showing displacement ellipsoids drawn at the 50% level. Symmetry code: 1 − *x*, 1 − *y*, 1 − *z*.

**Figure 2:**
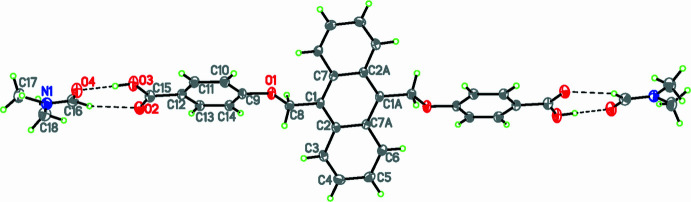
The mol­ecular structure of **2** showing displacement ellipsoids drawn at the 50% level. Symmetry code: 1 − *x*, −*y*, −*z*.

Compound **1** (Fig. 1 ▸) crystallizes in the monoclinic space group *P*2_1_/*c* with half of the main mol­ecule and two di­methyl­acetamide (DMA) solvent mol­ecules in the asymmetric unit. The dihedral angle between the anthracene ring system and the phenyl ring of the 2-mercapto­benzoic acid moiety is 71.43 (7)°. The torsion angle between the anthracene ring and phenyl group (C1—C8—S1—C9) is 178.13 (9)° indicating an *anti* conformation for this grouping and the C8—S1—C9 bond angle is 103.13 (7)°. Compound **2** (Fig. 2 ▸) crystallizes in space group *P*2_1_/*n* with half of the main mol­ecule and one di­methyl­formamide (DMF) mol­ecule in the asymmetric unit. The C8—O1—C9 bond angle is 117.8 (2)° and the C1—C8—O1—C9 torsion angle is −178.3 (2)°, indicating an *anti* conformation. The dihedral angle between the plane of anthracene ring system and the phenyl ring of the 4-hy­droxy­benzoic acid moiety is 75.27 (12)°.

## 3. Supra­molecular features

**Figure 3:**
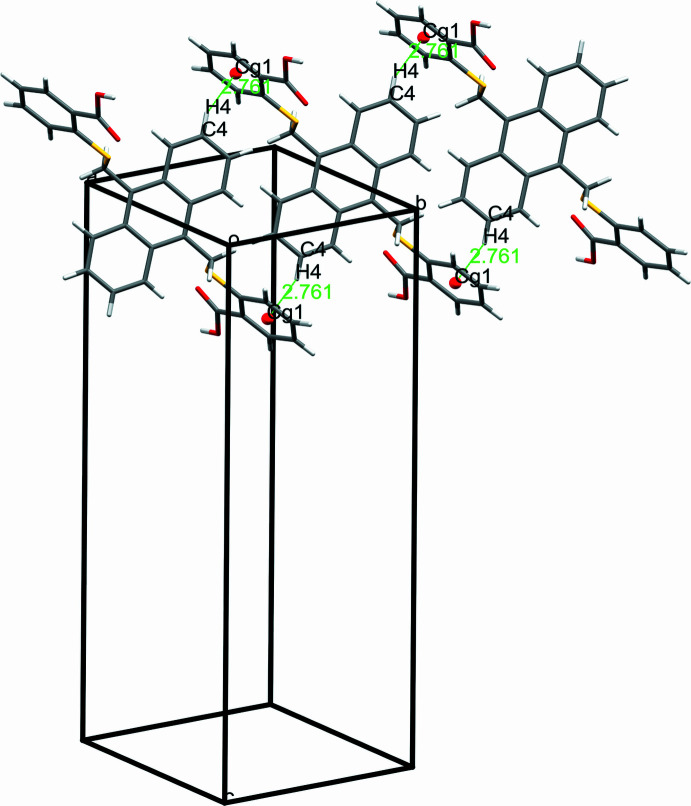
The packing of **1** showing C—H⋯π inter­actions.

In the extended structure of **1**, the carb­oxy­lic acid group of the ligand forms an O2—H2⋯O3*A* hydrogen bond with the DMA mol­ecule with an H⋯O bond length of 1.94 Å. The structure of **1** is further consolidated by a number of C—H⋯O, C—H⋯S and C—H⋯π weak inter­actions (Table 1 ▸). Among these, consecutive C—H⋯π inter­actions occur between the C4—H4 group of the anthracene ring and the centroid (*Cg*1) of the adjacent benzene ring with an H⋯π separation of 2.76 Å (Fig. 3 ▸). The packing also features a weak hydrogen bond between the methyl­ene hydrogen atoms of the anthracene ring and oxygen atom of DMF mol­ecule (C8—H8B⋯O4*A*) at a distance of 2.48 Å, forming infinite chains running parallel to each other.

**Figure 4:**
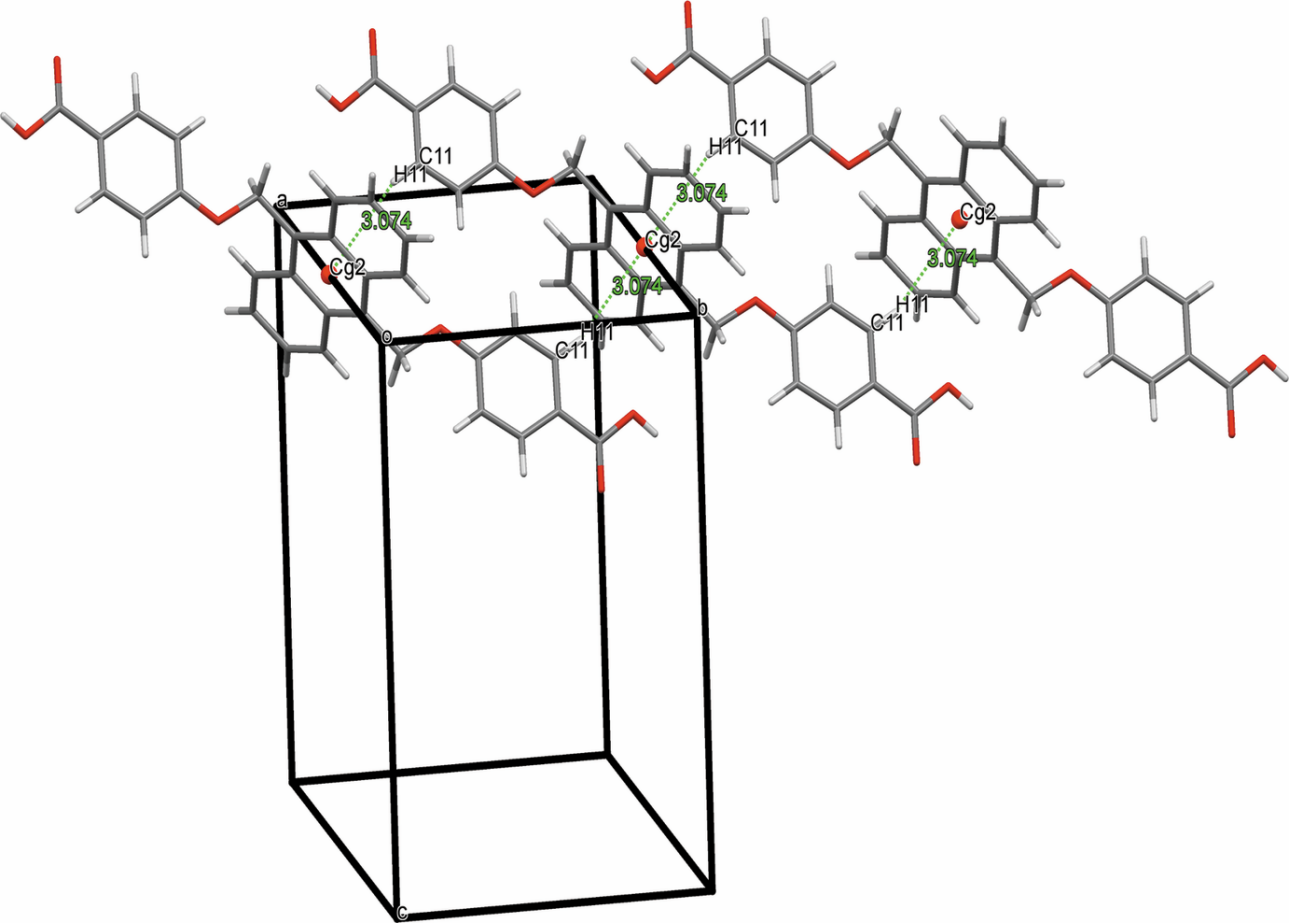
The packing of **2** showing C—H⋯π inter­actions.

A similar strong hydrogen bond occurs in **2**, between the carb­oxy­lic acid group of the ligand and oxygen atom of the DMF mol­ecule (O3—H3⋯O4) at a distance of 1.74 Å and the packing is supported by various weak inter­actions (Table 2 ▸), including consecutive C—H⋯π inter­actions involving the aromatic C11—H11 group of the phenyl ring and the centroid (*Cg2*) of the adjacent anthracene ring at an H⋯π distance of 3.07 Å, resulting in an array of chains running parallel to each other (Fig. 4 ▸). Additionally, the packing of **2** is reinforced by the hydrogen-bond inter­actions between the aromatic H atom of the benzene ring of one mol­ecule and the oxygen atom of the ether bond in the adjacent mol­ecule (C10—H10⋯O1) at a distance of 2.67 Å.

## 4. Database survey

A simple name search in the Cambridge Structural Database (CSD version 5.46, November 2024; Groom *et al.*, 2016 ▸) of both compounds **1** and **2** resulted in no hits. However, searching for the fragment anthracene-9,10-diylbis(methyl­ene) resulted in more than thirty similar structures, for example including CSD refcode BIHNIR (Suresh *et al.*, 2013 ▸), XUTHAZ (Verma *et al.*, 2025 ▸), TAPYEQ (Chen *et al.*, 2010 ▸), WUTGUO01 (Kan *et al.*, 2011 ▸), YIGCEA (Li *et al.*, 2023 ▸) and ZIHFEF (Verma *et al.*, 2023 ▸). These structures are either ligand mol­ecules or metal-organic structures having different substituents on the anthracene-9,10-diylbis(methyl­ene) moiety.

## 5. Synthesis and crystallization

**Figure 5:**
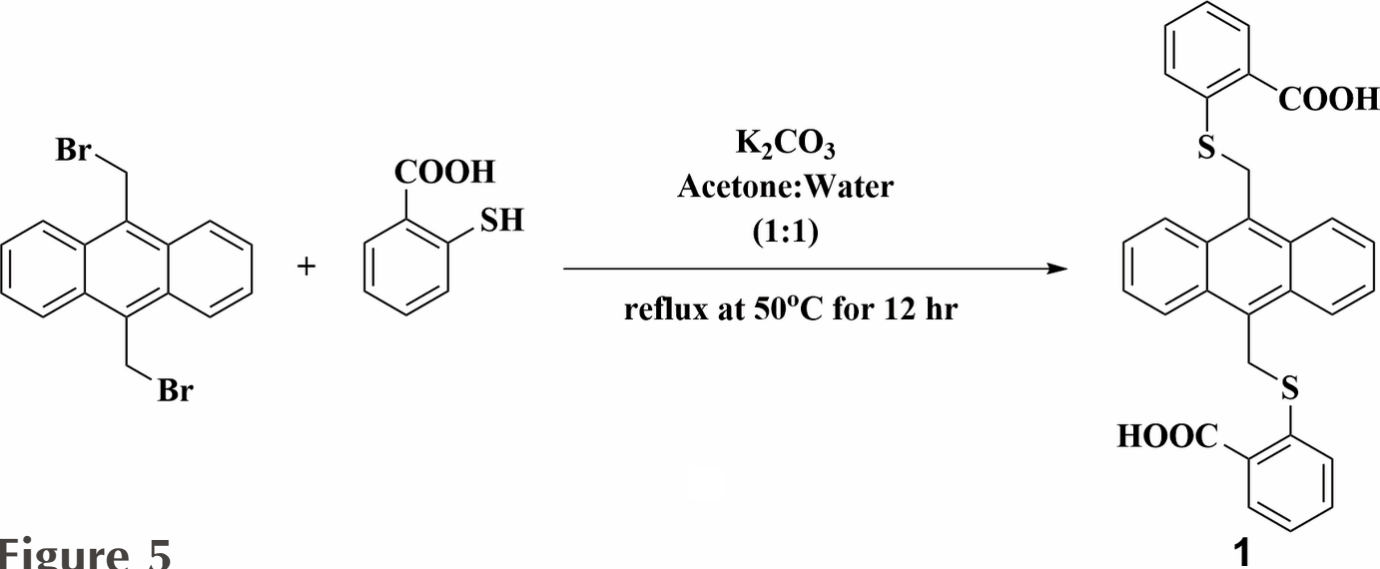
Synthesis schemes for compound **1**.

As outlined in Figs. 5 ▸ and 6 ▸, 2-mercapto­benzoic acid (2.0 mmol) and potassium carbonate (4.0 mmol) were dissolved in an acetone:water (1:1) mixture and refluxed for 1 h. After an hour, 9,10-bis­(bromo­meth­yl)anthracene (1.0 mmol) was added to the reaction mixture and it was refluxed overnight. After the completion of reaction, mixture was neutralized with 1 *N* HCl solution and the yellow precipitate was filtered, washed with water and dried to yield a yellow solid (yield: 92%). ^1^H-NMR (500MHz, DMSO-*d*_6_): *δ* 5.17 (*s*, 4H), 7.27–7.31 (*m*, 2H), 7.58–7.60 (*m*, 4H), 7.64–7.67 (*m*, 2H), 7.90–7.93 (*m*, 4H), 8.37–8.39 (*m*, 4H), 13.05 (*s*, 2H). The crystals of **1** were obtained by a solvothermal method using DMA solvent at 363 K for 96 h.

To prepare **2**, methyl-4-hy­droxy­benzoate was synthesized by following the previously reported procedure (Mondal *et al.*, 2023 ▸). Methyl-4-hy­droxy­benzoate (2.0 mmol) and potassium carbonate (2.2 mmol) were dissolved in aceto­nitrile (20 ml) and refluxed for 1 h. After an hour, 9,10-bis­(bromo­meth­yl)anthracene (1.0 mmol) was added to the reaction mixture and refluxed overnight. After the completion of reaction, it was neutralized with 1 *N* HCl solution and the solid product was filtered, washed with water and dried in a hot air oven (yield: 92%). ^1^H-NMR (500MHz, DMSO-*d*_6_): *δ* 3.81 (*s*, 6H), 6.17 (*s*, 4H), 7.27–7.29 (*d*, 4H), 7.59–7.61 (*m*, 4H), 7.95–7.96 (*d*, 4H), 8.39–8.41 (*m*, 4H). The methyl ester derivative was taken in a 100 ml round-bottom flask and dissolved in mixed solvents of THF:MeOH (1:1). 4 *M* NaOH was added into the reaction mixture and stirred for 24 h at RT. After the completion of reaction, it was neutralized with 1 *N* HCl solution and precipitate was filtered, washed with water and dried (yield: 85%). Crystals of **2** were obtained by a solvothermal method using DMF solvent at 373 K for 72 h.

## 6. Refinement

Crystal data, data collection and structure refinement details are summarized in Table 3 ▸. The H atoms attached to carbon atoms were refined using a riding model with *U*_iso_(H) = 1.2*U*_eq_(C). In **1**, both DMA mol­ecules were found to be disordered in a similar manner. This was modeled using SAME instructions in *SHELXL* for each component and resulted in occupancies of 0.799 (3)/0.201 (3) and 0.804 (3)/0.196 (3), respectively.

## Supplementary Material

Crystal structure: contains datablock(s) 1, 2. DOI: 10.1107/S2056989025008503/hb8146sup1.cif

Structure factors: contains datablock(s) 1. DOI: 10.1107/S2056989025008503/hb81461sup2.hkl

Structure factors: contains datablock(s) 2. DOI: 10.1107/S2056989025008503/hb81462sup3.hkl

CCDC references: 2491864, 2491863

Additional supporting information:  crystallographic information; 3D view; checkCIF report

## Figures and Tables

**Figure 6:**
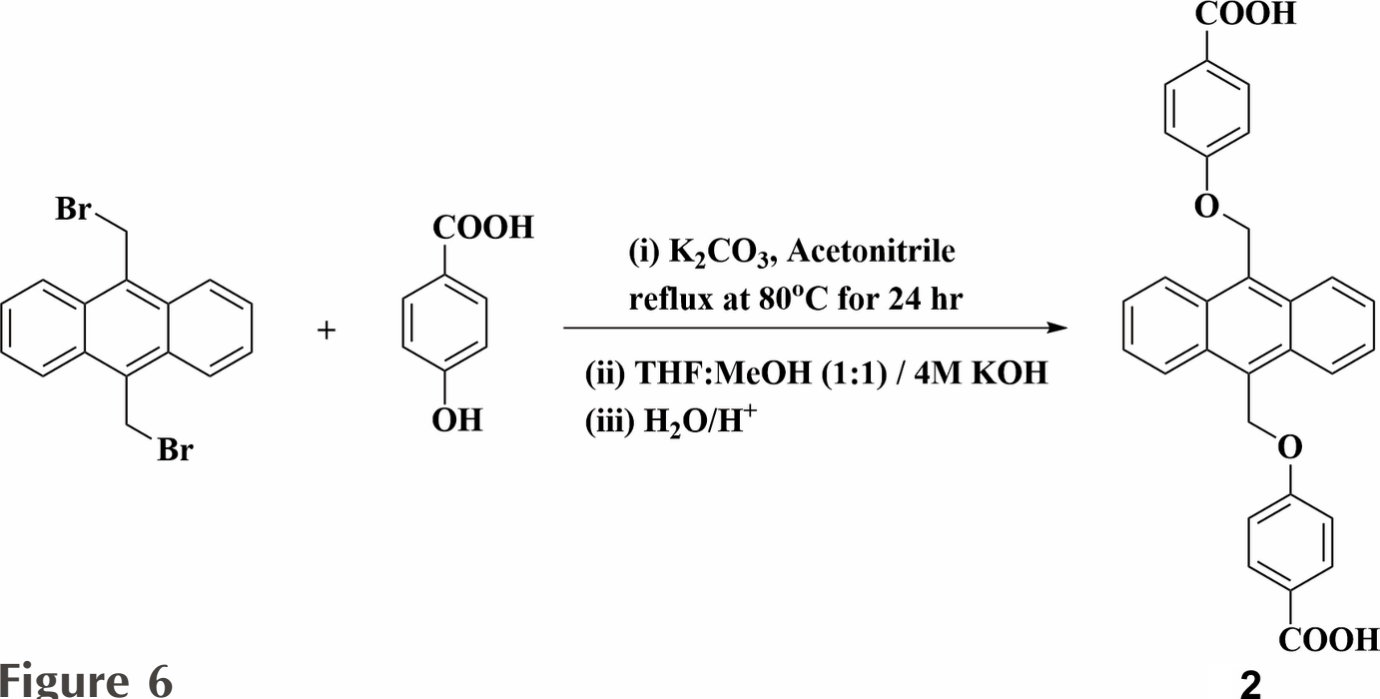
Synthesis schemes for compound **2**.

**Table 1:** Hydrogen-bond geometry (Å, °) for **1**[Chem scheme1] *Cg*1 is the centroid of the C9–C14 ring of **1**.

*D*—H⋯*A*	*D*—H	H⋯*A*	*D*⋯*A*	*D*—H⋯*A*
O2—H2⋯O3*A*^i^	0.84	1.94	2.770 (14)	169
C8—H8*B*⋯O4*A*^ii^	0.99	2.48	3.126 (8)	122
C17—H17*A*⋯O4^iii^	0.98	2.62	3.518 (8)	152
C18*A*—H18*D*⋯S1^iii^	0.98	3.02	3.83 (3)	142
C19*A*—H19*E*⋯O4*A*^iii^	0.98	2.43	3.40 (4)	168
C21—H21*C*⋯O1	0.98	2.49	3.422 (7)	158
C22*A*—H22*F*⋯O1	0.98	2.54	3.51 (3)	168
C4—H4⋯*Cg*1	0.95	2.76	3.49	135

Symmetry codes: (i) 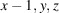; (ii) 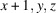; (iii) 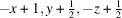.

**Table 2:** Hydrogen-bond geometry (Å, °) for **2**[Chem scheme1] *Cg*2 is the centroid of the C9–C14 ring of **2**.

*D*—H⋯*A*	*D*—H	H⋯*A*	*D*⋯*A*	*D*—H⋯*A*
O3—H3⋯O4	0.84	1.74	2.575 (3)	175
C8—H8*A*⋯O2^i^	0.99	2.54	3.429 (4)	149
C16—H16⋯O2	0.95	2.31	3.080 (4)	138
C4—H4⋯*Cg*2	0.95	3.07	3.96	157

Symmetry code: (i) 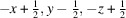.

**Table 3:** Experimental details

	**1**	**2**
Crystal data		
Chemical formula	C_30_H_22_O_4_S_2_·4C_4_H_9_NO	C_30_H_22_O_6_·2C_3_H_7_NO
*M*_r_	859.08	624.67
Crystal system, space group	Monoclinic, *P*2_1_/*c*	Monoclinic, *P*2_1_/*n*
Temperature (K)	104	104
*a*, *b*, *c* (Å)	9.9818 (3), 9.2026 (2), 24.1998 (7)	10.8761 (10), 9.6711 (9), 14.8968 (14)
β (°)	91.016 (1)	100.519 (3)
*V* (Å^3^)	2222.61 (10)	1540.6 (2)
*Z*	2	2
Radiation type	Mo *K*α	Mo *K*α
μ (mm^−1^)	0.18	0.10
Crystal size (mm)	0.35 × 0.15 × 0.12	0.26 × 0.19 × 0.16

Data collection		
Diffractometer	Bruker APEXII CCD	Bruker APEXII CCD
Absorption correction	Multi-scan (*OLEX2*; Dolomanov *et al.*, 2009 ▸)	Multi-scan (*OLEX2*; Dolomanov *et al.*, 2009 ▸)
*T*_min_, *T*_max_	0.706, 0.746	0.666, 0.745
No. of measured, independent and observed [*I* > 2σ(*I*)] reflections	84923, 5544, 4374	49734, 3169, 1669
*R*_int_	0.076	0.179
(sin θ/λ)_max_ (Å^−1^)	0.668	0.627

Refinement		
*R*[*F*^2^ > 2σ(*F*^2^)], *wR*(*F*^2^), *S*	0.045, 0.112, 1.03	0.069, 0.182, 1.02
No. of reflections	5544	3169
No. of parameters	394	211
No. of restraints	422	0
H-atom treatment	H-atom parameters constrained	H-atom parameters constrained
Δρ_max_, Δρ_min_ (e Å^−3^)	0.41, −0.27	0.31, −0.24

Computer programs: *APEX4*, *APEX2* and *SAINT* (Bruker, 2016 ▸), *SHELXT2018/2* (Sheldrick, 2015*a* ▸), *SHELXL-2019/2* (Sheldrick, 2015*b* ▸) and *OLEX2* (Dolomanov *et al.*, 2009 ▸).

## supplementary crystallographic information

### 2,2'-{[Anthracene-9,10-diylbis(methylene)]bis(sulfanediyl)}dibenzoic acid dimethylacetamide tetrasolvate (1) Crystal data

**Table:**

C_30_H_22_O_4_S_2_·4C_4_H_9_NO	*F*(000) = 916
*M**_r_* = 859.08	*D*_x_ = 1.284 Mg m^−^^3^
Monoclinic, *P*2_1_/*c*	Mo *K*α radiation, λ = 0.71073 Å
*a* = 9.9818 (3) Å	Cell parameters from 9910 reflections
*b* = 9.2026 (2) Å	θ = 2.4–25.7°
*c* = 24.1998 (7) Å	µ = 0.18 mm^−^^1^
β = 91.016 (1)°	*T* = 104 K
*V* = 2222.61 (10) Å^3^	Block, clear yellowish yellow
*Z* = 2	0.35 × 0.15 × 0.12 mm

### 2,2'-{[Anthracene-9,10-diylbis(methylene)]bis(sulfanediyl)}dibenzoic acid dimethylacetamide tetrasolvate (1) Data collection

**Table:**

Bruker APEXII CCD diffractometer	4374 reflections with *I* > 2σ(*I*)
ω and φ scans	*R*_int_ = 0.076
Absorption correction: multi-scan (Olex2; Dolomanov *et al.*, 2009)	θ_max_ = 28.3°, θ_min_ = 1.7°
*T*_min_ = 0.706, *T*_max_ = 0.746	*h* = −13→13
84923 measured reflections	*k* = −12→12
5544 independent reflections	*l* = −32→32

### 2,2'-{[Anthracene-9,10-diylbis(methylene)]bis(sulfanediyl)}dibenzoic acid dimethylacetamide tetrasolvate (1) Refinement

**Table:**

Refinement on *F*^2^	Primary atom site location: dual
Least-squares matrix: full	Hydrogen site location: inferred from neighbouring sites
*R*[*F*^2^ > 2σ(*F*^2^)] = 0.045	H-atom parameters constrained
*wR*(*F*^2^) = 0.112	*w* = 1/[σ^2^(*F*_o_^2^) + (0.0474*P*)^2^ + 1.119*P*] where *P* = (*F*_o_^2^ + 2*F*_c_^2^)/3
*S* = 1.03	(Δ/σ)_max_ = 0.001
5544 reflections	Δρ_max_ = 0.41 e Å^−^^3^
394 parameters	Δρ_min_ = −0.27 e Å^−^^3^
422 restraints	

### 2,2'-{[Anthracene-9,10-diylbis(methylene)]bis(sulfanediyl)}dibenzoic acid dimethylacetamide tetrasolvate (1) Special details

**Table:**

Geometry. All esds (except the esd in the dihedral angle between two l.s. planes) are estimated using the full covariance matrix. The cell esds are taken into account individually in the estimation of esds in distances, angles and torsion angles; correlations between esds in cell parameters are only used when they are defined by crystal symmetry. An approximate (isotropic) treatment of cell esds is used for estimating esds involving l.s. planes.

### 2,2'-{[Anthracene-9,10-diylbis(methylene)]bis(sulfanediyl)}dibenzoic acid dimethylacetamide tetrasolvate (1) Fractional atomic coordinates and isotropic or equivalent isotropic displacement parameters (Å^2^)

**Table:**

	*x*	*y*	*z*	*U*_iso_*/*U*_eq_	Occ. (<1)
S1	0.49737 (4)	0.76829 (4)	0.38089 (2)	0.01827 (10)	
O1	0.29231 (11)	0.87413 (12)	0.31863 (4)	0.0263 (3)	
O2	0.31257 (12)	0.96903 (14)	0.23437 (5)	0.0310 (3)	
H2	0.230729	0.948146	0.232140	0.047*	
C1	0.56861 (14)	0.58987 (15)	0.46196 (6)	0.0167 (3)	
C2	0.52491 (14)	0.45046 (15)	0.44587 (6)	0.0173 (3)	
C3	0.54843 (16)	0.39496 (16)	0.39171 (6)	0.0224 (3)	
H3	0.593487	0.454054	0.365752	0.027*	
C4	0.50773 (17)	0.25944 (17)	0.37653 (6)	0.0267 (4)	
H4	0.525167	0.224961	0.340360	0.032*	
C5	0.43976 (17)	0.16965 (17)	0.41413 (7)	0.0273 (4)	
H5	0.411335	0.075326	0.403109	0.033*	
C6	0.41483 (16)	0.21774 (16)	0.46609 (6)	0.0228 (3)	
H6	0.369268	0.155798	0.490923	0.027*	
C7	0.54455 (14)	0.64077 (15)	0.51567 (6)	0.0171 (3)	
C8	0.63542 (15)	0.68688 (16)	0.42042 (6)	0.0192 (3)	
H8A	0.694092	0.629672	0.396056	0.023*	
H8B	0.689817	0.762652	0.439262	0.023*	
C9	0.57997 (15)	0.88537 (15)	0.33438 (6)	0.0177 (3)	
C10	0.71660 (16)	0.91600 (16)	0.34057 (6)	0.0217 (3)	
H10	0.766922	0.871749	0.369702	0.026*	
C11	0.77937 (17)	1.01029 (17)	0.30463 (7)	0.0251 (3)	
H11	0.871972	1.031090	0.309790	0.030*	
C12	0.70906 (17)	1.07443 (16)	0.26140 (6)	0.0247 (3)	
H12	0.752673	1.139489	0.237117	0.030*	
C13	0.57494 (16)	1.04298 (16)	0.25390 (6)	0.0223 (3)	
H13	0.526989	1.085006	0.223637	0.027*	
C14	0.50800 (15)	0.95009 (15)	0.29011 (6)	0.0195 (3)	
C15	0.36146 (16)	0.92700 (16)	0.28293 (6)	0.0211 (3)	
O3	1.0671 (4)	0.9058 (3)	0.2180 (2)	0.0353 (7)	0.799 (3)
N1	0.88738 (18)	0.9245 (2)	0.16135 (7)	0.0285 (5)	0.799 (3)
C16	0.9981 (2)	0.9800 (2)	0.18493 (9)	0.0274 (5)	0.799 (3)
C17	1.0361 (8)	1.1340 (7)	0.1719 (4)	0.0353 (10)	0.799 (3)
H17A	1.050112	1.143580	0.132038	0.053*	0.799 (3)
H17B	1.118931	1.159241	0.191974	0.053*	0.799 (3)
H17C	0.964103	1.199522	0.183008	0.053*	0.799 (3)
C18	0.8425 (6)	0.7782 (5)	0.1765 (3)	0.0369 (10)	0.799 (3)
H18A	0.816616	0.724555	0.142961	0.055*	0.799 (3)
H18B	0.765414	0.785502	0.200811	0.055*	0.799 (3)
H18C	0.915606	0.726820	0.195702	0.055*	0.799 (3)
C19	0.7988 (6)	1.0049 (7)	0.1234 (3)	0.0360 (11)	0.799 (3)
H19A	0.781855	0.946712	0.090114	0.054*	0.799 (3)
H19B	0.841408	1.096737	0.113248	0.054*	0.799 (3)
H19C	0.713733	1.024955	0.141564	0.054*	0.799 (3)
O3A	1.0538 (16)	0.8659 (15)	0.2192 (8)	0.036 (2)	0.201 (3)
N1A	0.9312 (8)	1.0021 (9)	0.1604 (3)	0.0294 (12)	0.201 (3)
C16A	0.9542 (8)	0.8815 (9)	0.1884 (3)	0.0296 (12)	0.201 (3)
C17A	0.858 (2)	0.757 (2)	0.1825 (13)	0.035 (3)	0.201 (3)
H17D	0.766243	0.793275	0.184933	0.052*	0.201 (3)
H17E	0.875504	0.686684	0.212185	0.052*	0.201 (3)
H17F	0.870396	0.709975	0.146647	0.052*	0.201 (3)
C18A	0.815 (2)	1.025 (3)	0.1230 (13)	0.034 (3)	0.201 (3)
H18D	0.767338	1.113996	0.133881	0.051*	0.201 (3)
H18E	0.753673	0.942141	0.125383	0.051*	0.201 (3)
H18F	0.845163	1.035463	0.084941	0.051*	0.201 (3)
C19A	1.022 (3)	1.128 (3)	0.1644 (16)	0.033 (3)	0.201 (3)
H19D	0.983823	1.201294	0.188723	0.050*	0.201 (3)
H19E	1.034642	1.169307	0.127565	0.050*	0.201 (3)
H19F	1.109197	1.096129	0.179621	0.050*	0.201 (3)
O4	−0.0836 (2)	0.7997 (3)	0.45732 (9)	0.0501 (6)	0.804 (3)
N2	0.1140 (2)	0.7946 (2)	0.50291 (9)	0.0326 (5)	0.804 (3)
C20	0.0317 (2)	0.8450 (2)	0.46303 (9)	0.0317 (5)	0.804 (3)
C21	0.0868 (7)	0.9608 (6)	0.4254 (2)	0.0367 (8)	0.804 (3)
H21A	0.123902	1.040534	0.447754	0.055*	0.804 (3)
H21B	0.014736	0.997995	0.401298	0.055*	0.804 (3)
H21C	0.157583	0.918999	0.402787	0.055*	0.804 (3)
C22	0.0711 (7)	0.6745 (7)	0.53909 (16)	0.0553 (13)	0.804 (3)
H22A	0.083561	0.703059	0.577852	0.083*	0.804 (3)
H22B	0.125009	0.587919	0.531637	0.083*	0.804 (3)
H22C	−0.023695	0.652905	0.531759	0.083*	0.804 (3)
C23	0.2478 (4)	0.8572 (5)	0.51334 (18)	0.0365 (9)	0.804 (3)
H23A	0.300280	0.791520	0.537132	0.055*	0.804 (3)
H23B	0.238720	0.951551	0.531667	0.055*	0.804 (3)
H23C	0.293469	0.870156	0.478167	0.055*	0.804 (3)
O4A	−0.0743 (8)	0.7207 (12)	0.4693 (4)	0.0496 (19)	0.196 (3)
N2A	0.1243 (8)	0.8380 (9)	0.4771 (4)	0.0332 (12)	0.196 (3)
C20A	0.0371 (9)	0.7362 (11)	0.4923 (4)	0.0369 (12)	0.196 (3)
C21A	0.064 (3)	0.694 (3)	0.5513 (6)	0.039 (2)	0.196 (3)
H21D	0.129391	0.761244	0.567808	0.059*	0.196 (3)
H21E	0.099197	0.594970	0.552752	0.059*	0.196 (3)
H21F	−0.019839	0.699037	0.571807	0.059*	0.196 (3)
C22A	0.083 (3)	0.933 (3)	0.4313 (10)	0.035 (2)	0.196 (3)
H22D	0.102444	1.034030	0.440877	0.053*	0.196 (3)
H22E	−0.013882	0.921667	0.424344	0.053*	0.196 (3)
H22F	0.131343	0.905840	0.398021	0.053*	0.196 (3)
C23A	0.2596 (15)	0.836 (3)	0.5004 (8)	0.040 (3)	0.196 (3)
H23D	0.320334	0.885391	0.475125	0.060*	0.196 (3)
H23E	0.288775	0.735411	0.505731	0.060*	0.196 (3)
H23F	0.260857	0.886607	0.536101	0.060*	0.196 (3)

### 2,2'-{[Anthracene-9,10-diylbis(methylene)]bis(sulfanediyl)}dibenzoic acid dimethylacetamide tetrasolvate (1) Atomic displacement parameters (Å^2^)

**Table:**

	*U*^11^	*U*^22^	*U*^33^	*U*^12^	*U*^13^	*U*^23^
S1	0.0203 (2)	0.01784 (18)	0.01676 (18)	−0.00194 (14)	0.00182 (13)	0.00343 (13)
O1	0.0255 (6)	0.0321 (6)	0.0213 (6)	0.0003 (5)	0.0022 (5)	0.0035 (5)
O2	0.0273 (6)	0.0448 (7)	0.0209 (6)	0.0008 (5)	−0.0034 (5)	0.0087 (5)
C1	0.0164 (7)	0.0172 (7)	0.0166 (7)	0.0000 (5)	−0.0002 (5)	0.0038 (5)
C2	0.0179 (7)	0.0180 (7)	0.0161 (7)	0.0015 (5)	0.0010 (5)	0.0016 (5)
C3	0.0270 (8)	0.0231 (7)	0.0171 (7)	0.0010 (6)	0.0038 (6)	0.0013 (6)
C4	0.0355 (9)	0.0251 (8)	0.0196 (8)	0.0025 (7)	0.0030 (7)	−0.0056 (6)
C5	0.0334 (9)	0.0191 (7)	0.0293 (8)	−0.0021 (7)	0.0003 (7)	−0.0062 (6)
C6	0.0270 (8)	0.0180 (7)	0.0235 (8)	−0.0031 (6)	0.0029 (6)	0.0014 (6)
C7	0.0176 (7)	0.0161 (7)	0.0177 (7)	0.0009 (5)	−0.0002 (5)	0.0006 (5)
C8	0.0203 (8)	0.0197 (7)	0.0176 (7)	−0.0021 (6)	0.0019 (6)	0.0035 (6)
C9	0.0246 (8)	0.0144 (6)	0.0144 (7)	−0.0005 (6)	0.0046 (6)	0.0001 (5)
C10	0.0251 (8)	0.0206 (7)	0.0193 (7)	0.0002 (6)	0.0024 (6)	0.0030 (6)
C11	0.0246 (8)	0.0245 (8)	0.0264 (8)	−0.0031 (6)	0.0068 (7)	0.0019 (6)
C12	0.0337 (9)	0.0196 (7)	0.0211 (8)	−0.0006 (6)	0.0098 (7)	0.0030 (6)
C13	0.0333 (9)	0.0184 (7)	0.0153 (7)	0.0030 (6)	0.0047 (6)	0.0007 (5)
C14	0.0268 (8)	0.0169 (7)	0.0151 (7)	0.0013 (6)	0.0037 (6)	−0.0023 (5)
C15	0.0280 (8)	0.0181 (7)	0.0171 (7)	0.0030 (6)	0.0006 (6)	−0.0015 (6)
O3	0.0269 (12)	0.0420 (17)	0.0365 (11)	−0.0029 (11)	−0.0087 (9)	0.0098 (13)
N1	0.0231 (9)	0.0343 (10)	0.0281 (9)	0.0025 (8)	−0.0027 (7)	−0.0040 (8)
C16	0.0213 (10)	0.0370 (11)	0.0240 (10)	0.0014 (8)	0.0025 (8)	−0.0010 (8)
C17	0.033 (3)	0.0390 (15)	0.034 (3)	−0.0047 (13)	0.0030 (16)	0.0056 (16)
C18	0.0321 (19)	0.0303 (16)	0.048 (2)	0.0022 (14)	−0.0077 (14)	−0.0056 (14)
C19	0.0286 (19)	0.048 (2)	0.0311 (15)	0.0058 (17)	−0.0070 (13)	0.0038 (15)
O3A	0.027 (4)	0.042 (5)	0.037 (4)	0.000 (4)	−0.005 (3)	0.007 (4)
N1A	0.022 (2)	0.038 (2)	0.029 (2)	0.001 (2)	−0.001 (2)	0.001 (2)
C16A	0.023 (2)	0.037 (2)	0.029 (2)	0.002 (2)	0.002 (2)	−0.002 (2)
C17A	0.030 (5)	0.034 (4)	0.040 (5)	−0.002 (4)	−0.002 (4)	−0.005 (4)
C18A	0.022 (4)	0.043 (5)	0.036 (5)	−0.003 (4)	−0.008 (4)	0.001 (4)
C19A	0.024 (5)	0.042 (4)	0.033 (6)	−0.005 (4)	0.001 (4)	0.003 (4)
O4	0.0313 (10)	0.0784 (17)	0.0403 (11)	−0.0231 (11)	−0.0036 (8)	−0.0016 (11)
N2	0.0279 (10)	0.0325 (10)	0.0374 (11)	−0.0028 (8)	0.0053 (8)	0.0029 (8)
C20	0.0284 (11)	0.0387 (11)	0.0282 (10)	−0.0058 (9)	0.0042 (8)	−0.0080 (9)
C21	0.0410 (15)	0.040 (2)	0.0293 (17)	−0.0050 (15)	0.0059 (13)	0.0024 (12)
C22	0.056 (2)	0.070 (3)	0.040 (2)	−0.0181 (19)	0.0098 (19)	0.0103 (19)
C23	0.0215 (12)	0.0405 (18)	0.047 (2)	0.0020 (11)	−0.0001 (13)	−0.0004 (14)
O4A	0.033 (3)	0.063 (4)	0.052 (4)	−0.013 (3)	−0.004 (3)	−0.001 (4)
N2A	0.029 (2)	0.040 (2)	0.031 (2)	−0.001 (2)	0.004 (2)	0.001 (2)
C20A	0.032 (2)	0.045 (2)	0.034 (2)	−0.005 (2)	0.004 (2)	−0.002 (2)
C21A	0.043 (5)	0.037 (5)	0.038 (4)	−0.003 (4)	0.008 (4)	0.008 (4)
C22A	0.035 (5)	0.040 (5)	0.030 (5)	0.004 (4)	0.002 (4)	−0.005 (3)
C23A	0.028 (4)	0.051 (5)	0.042 (5)	0.002 (4)	0.005 (4)	0.003 (5)

### 2,2'-{[Anthracene-9,10-diylbis(methylene)]bis(sulfanediyl)}dibenzoic acid dimethylacetamide tetrasolvate (1) Geometric parameters (Å, º)

**Table:**

S1—C9	1.7721 (14)	C19—H19C	0.9800
S1—C8	1.8245 (15)	O3A—C16A	1.241 (12)
O1—C15	1.2169 (18)	N1A—C16A	1.318 (10)
O2—C15	1.3222 (18)	N1A—C19A	1.473 (14)
O2—H2	0.8400	N1A—C18A	1.477 (14)
C1—C7	1.406 (2)	C16A—C17A	1.497 (14)
C1—C2	1.408 (2)	C17A—H17D	0.9800
C1—C8	1.5086 (19)	C17A—H17E	0.9800
C2—C3	1.430 (2)	C17A—H17F	0.9800
C2—C7^i^	1.4405 (19)	C18A—H18D	0.9800
C3—C4	1.360 (2)	C18A—H18E	0.9800
C3—H3	0.9500	C18A—H18F	0.9800
C4—C5	1.412 (2)	C19A—H19D	0.9800
C4—H4	0.9500	C19A—H19E	0.9800
C5—C6	1.360 (2)	C19A—H19F	0.9800
C5—H5	0.9500	O4—C20	1.229 (3)
C6—C7^i^	1.431 (2)	N2—C20	1.339 (3)
C6—H6	0.9500	N2—C23	1.472 (4)
C8—H8A	0.9900	N2—C22	1.478 (6)
C8—H8B	0.9900	C20—C21	1.511 (4)
C9—C10	1.398 (2)	C21—H21A	0.9800
C9—C14	1.411 (2)	C21—H21B	0.9800
C10—C11	1.386 (2)	C21—H21C	0.9800
C10—H10	0.9500	C22—H22A	0.9800
C11—C12	1.382 (2)	C22—H22B	0.9800
C11—H11	0.9500	C22—H22C	0.9800
C12—C13	1.379 (2)	C23—H23A	0.9800
C12—H12	0.9500	C23—H23B	0.9800
C13—C14	1.402 (2)	C23—H23C	0.9800
C13—H13	0.9500	O4A—C20A	1.243 (10)
C14—C15	1.485 (2)	N2A—C20A	1.335 (10)
O3—C16	1.249 (4)	N2A—C23A	1.454 (13)
N1—C16	1.336 (3)	N2A—C22A	1.466 (13)
N1—C19	1.464 (5)	C20A—C21A	1.497 (13)
N1—C18	1.467 (5)	C21A—H21D	0.9800
C16—C17	1.502 (5)	C21A—H21E	0.9800
C17—H17A	0.9800	C21A—H21F	0.9800
C17—H17B	0.9800	C22A—H22D	0.9800
C17—H17C	0.9800	C22A—H22E	0.9800
C18—H18A	0.9800	C22A—H22F	0.9800
C18—H18B	0.9800	C23A—H23D	0.9800
C18—H18C	0.9800	C23A—H23E	0.9800
C19—H19A	0.9800	C23A—H23F	0.9800
C19—H19B	0.9800		
			
C9—S1—C8	103.13 (7)	H19B—C19—H19C	109.5
C15—O2—H2	109.5	C16A—N1A—C19A	121.7 (14)
C7—C1—C2	120.14 (12)	C16A—N1A—C18A	124.4 (12)
C7—C1—C8	120.23 (13)	C19A—N1A—C18A	114.0 (16)
C2—C1—C8	119.57 (13)	O3A—C16A—N1A	122.5 (10)
C1—C2—C3	121.59 (13)	O3A—C16A—C17A	118.2 (12)
C1—C2—C7^i^	120.19 (13)	N1A—C16A—C17A	119.4 (11)
C3—C2—C7^i^	118.22 (13)	C16A—C17A—H17D	109.5
C4—C3—C2	121.46 (14)	C16A—C17A—H17E	109.5
C4—C3—H3	119.3	H17D—C17A—H17E	109.5
C2—C3—H3	119.3	C16A—C17A—H17F	109.5
C3—C4—C5	120.48 (14)	H17D—C17A—H17F	109.5
C3—C4—H4	119.8	H17E—C17A—H17F	109.5
C5—C4—H4	119.8	N1A—C18A—H18D	109.5
C6—C5—C4	120.22 (14)	N1A—C18A—H18E	109.5
C6—C5—H5	119.9	H18D—C18A—H18E	109.5
C4—C5—H5	119.9	N1A—C18A—H18F	109.5
C5—C6—C7^i^	121.72 (14)	H18D—C18A—H18F	109.5
C5—C6—H6	119.1	H18E—C18A—H18F	109.5
C7^i^—C6—H6	119.1	N1A—C19A—H19D	109.5
C1—C7—C6^i^	122.42 (13)	N1A—C19A—H19E	109.5
C1—C7—C2^i^	119.68 (13)	H19D—C19A—H19E	109.5
C6^i^—C7—C2^i^	117.90 (13)	N1A—C19A—H19F	109.5
C1—C8—S1	104.70 (10)	H19D—C19A—H19F	109.5
C1—C8—H8A	110.8	H19E—C19A—H19F	109.5
S1—C8—H8A	110.8	C20—N2—C23	122.1 (3)
C1—C8—H8B	110.8	C20—N2—C22	120.4 (3)
S1—C8—H8B	110.8	C23—N2—C22	117.5 (4)
H8A—C8—H8B	108.9	O4—C20—N2	121.6 (2)
C10—C9—C14	118.61 (13)	O4—C20—C21	121.4 (3)
C10—C9—S1	121.23 (11)	N2—C20—C21	116.9 (3)
C14—C9—S1	120.16 (11)	C20—C21—H21A	109.5
C11—C10—C9	120.66 (14)	C20—C21—H21B	109.5
C11—C10—H10	119.7	H21A—C21—H21B	109.5
C9—C10—H10	119.7	C20—C21—H21C	109.5
C12—C11—C10	120.88 (15)	H21A—C21—H21C	109.5
C12—C11—H11	119.6	H21B—C21—H21C	109.5
C10—C11—H11	119.6	N2—C22—H22A	109.5
C13—C12—C11	119.28 (14)	N2—C22—H22B	109.5
C13—C12—H12	120.4	H22A—C22—H22B	109.5
C11—C12—H12	120.4	N2—C22—H22C	109.5
C12—C13—C14	121.20 (14)	H22A—C22—H22C	109.5
C12—C13—H13	119.4	H22B—C22—H22C	109.5
C14—C13—H13	119.4	N2—C23—H23A	109.5
C13—C14—C9	119.34 (14)	N2—C23—H23B	109.5
C13—C14—C15	119.55 (13)	H23A—C23—H23B	109.5
C9—C14—C15	121.04 (13)	N2—C23—H23C	109.5
O1—C15—O2	122.88 (15)	H23A—C23—H23C	109.5
O1—C15—C14	122.95 (14)	H23B—C23—H23C	109.5
O2—C15—C14	114.17 (13)	C20A—N2A—C23A	119.4 (12)
C16—N1—C19	124.1 (3)	C20A—N2A—C22A	116.6 (11)
C16—N1—C18	119.9 (3)	C23A—N2A—C22A	123.3 (14)
C19—N1—C18	115.9 (3)	O4A—C20A—N2A	122.7 (9)
O3—C16—N1	120.5 (2)	O4A—C20A—C21A	122.7 (13)
O3—C16—C17	120.8 (3)	N2A—C20A—C21A	109.7 (12)
N1—C16—C17	118.7 (3)	C20A—C21A—H21D	109.5
C16—C17—H17A	109.5	C20A—C21A—H21E	109.5
C16—C17—H17B	109.5	H21D—C21A—H21E	109.5
H17A—C17—H17B	109.5	C20A—C21A—H21F	109.5
C16—C17—H17C	109.5	H21D—C21A—H21F	109.5
H17A—C17—H17C	109.5	H21E—C21A—H21F	109.5
H17B—C17—H17C	109.5	N2A—C22A—H22D	109.5
N1—C18—H18A	109.5	N2A—C22A—H22E	109.5
N1—C18—H18B	109.5	H22D—C22A—H22E	109.5
H18A—C18—H18B	109.5	N2A—C22A—H22F	109.5
N1—C18—H18C	109.5	H22D—C22A—H22F	109.5
H18A—C18—H18C	109.5	H22E—C22A—H22F	109.5
H18B—C18—H18C	109.5	N2A—C23A—H23D	109.5
N1—C19—H19A	109.5	N2A—C23A—H23E	109.5
N1—C19—H19B	109.5	H23D—C23A—H23E	109.5
H19A—C19—H19B	109.5	N2A—C23A—H23F	109.5
N1—C19—H19C	109.5	H23D—C23A—H23F	109.5
H19A—C19—H19C	109.5	H23E—C23A—H23F	109.5
			
C7—C1—C2—C3	−179.61 (14)	C10—C9—C14—C13	−0.2 (2)
C8—C1—C2—C3	3.3 (2)	S1—C9—C14—C13	179.70 (11)
C7—C1—C2—C7^i^	−0.1 (2)	C10—C9—C14—C15	−177.18 (13)
C8—C1—C2—C7^i^	−177.13 (13)	S1—C9—C14—C15	2.68 (19)
C1—C2—C3—C4	179.07 (15)	C13—C14—C15—O1	−164.05 (14)
C7^i^—C2—C3—C4	−0.5 (2)	C9—C14—C15—O1	13.0 (2)
C2—C3—C4—C5	0.4 (2)	C13—C14—C15—O2	15.89 (19)
C3—C4—C5—C6	−0.3 (3)	C9—C14—C15—O2	−167.10 (13)
C4—C5—C6—C7^i^	0.2 (3)	C19—N1—C16—O3	178.2 (5)
C2—C1—C7—C6^i^	−179.13 (14)	C18—N1—C16—O3	3.2 (6)
C8—C1—C7—C6^i^	−2.1 (2)	C19—N1—C16—C17	−0.5 (7)
C2—C1—C7—C2^i^	0.1 (2)	C18—N1—C16—C17	−175.6 (6)
C8—C1—C7—C2^i^	177.11 (13)	C19A—N1A—C16A—O3A	−1 (3)
C7—C1—C8—S1	−95.52 (14)	C18A—N1A—C16A—O3A	180 (2)
C2—C1—C8—S1	81.56 (14)	C19A—N1A—C16A—C17A	−180 (3)
C9—S1—C8—C1	178.13 (9)	C18A—N1A—C16A—C17A	0 (3)
C8—S1—C9—C10	−11.00 (14)	C23—N2—C20—O4	−174.3 (3)
C8—S1—C9—C14	169.14 (11)	C22—N2—C20—O4	4.3 (4)
C14—C9—C10—C11	1.3 (2)	C23—N2—C20—C21	5.4 (5)
S1—C9—C10—C11	−178.57 (12)	C22—N2—C20—C21	−175.9 (4)
C9—C10—C11—C12	−1.0 (2)	C23A—N2A—C20A—O4A	−171.1 (13)
C10—C11—C12—C13	−0.5 (2)	C22A—N2A—C20A—O4A	0 (2)
C11—C12—C13—C14	1.6 (2)	C23A—N2A—C20A—C21A	33.2 (17)
C12—C13—C14—C9	−1.3 (2)	C22A—N2A—C20A—C21A	−155.7 (19)
C12—C13—C14—C15	175.77 (13)		

Symmetry code: (i) −*x*+1, −*y*+1, −*z*+1.

### 2,2'-{[Anthracene-9,10-diylbis(methylene)]bis(sulfanediyl)}dibenzoic acid dimethylacetamide tetrasolvate (1) Hydrogen-bond geometry (Å, º)

*Cg*1 is the centroid of the C9–C14 ring of (1).

**Table:**

*D*—H···*A*	*D*—H	H···*A*	*D*···*A*	*D*—H···*A*
O2—H2···O3*A*^ii^	0.84	1.94	2.770 (14)	169
C8—H8*B*···O4*A*^iii^	0.99	2.48	3.126 (8)	122
C17—H17*A*···O4^iv^	0.98	2.62	3.518 (8)	152
C18*A*—H18*D*···S1^iv^	0.98	3.02	3.83 (3)	142
C19*A*—H19*E*···O4*A*^iv^	0.98	2.43	3.40 (4)	168
C21—H21*C*···O1	0.98	2.49	3.422 (7)	158
C22*A*—H22*F*···O1	0.98	2.54	3.51 (3)	168
C4—H4···*Cg*1	0.95	2.76	3.49	135

Symmetry codes: (ii) *x*−1, *y*, *z*; (iii) *x*+1, *y*, *z*; (iv) −*x*+1, *y*+1/2, −*z*+1/2.

### 4,4'-{[Anthracene-9,10-diylbis(methylene)]bis(oxy)}dibenzoic acid dimethylformamide disolvate (2) Crystal data

**Table:**

C_30_H_22_O_6_·2C_3_H_7_NO	*F*(000) = 660
*M**_r_* = 624.67	*D*_x_ = 1.347 Mg m^−^^3^
Monoclinic, *P*2_1_/*n*	Mo *K*α radiation, λ = 0.71073 Å
*a* = 10.8761 (10) Å	Cell parameters from 1406 reflections
*b* = 9.6711 (9) Å	θ = 2.6–18.7°
*c* = 14.8968 (14) Å	µ = 0.10 mm^−^^1^
β = 100.519 (3)°	*T* = 104 K
*V* = 1540.6 (2) Å^3^	Plate, clear yellowish yellow
*Z* = 2	0.26 × 0.19 × 0.16 mm

### 4,4'-{[Anthracene-9,10-diylbis(methylene)]bis(oxy)}dibenzoic acid dimethylformamide disolvate (2) Data collection

**Table:**

Bruker APEXII CCD diffractometer	1669 reflections with *I* > 2σ(*I*)
φ and ω scans	*R*_int_ = 0.179
Absorption correction: multi-scan (Olex2; Dolomanov *et al.*, 2009)	θ_max_ = 26.5°, θ_min_ = 2.1°
*T*_min_ = 0.666, *T*_max_ = 0.745	*h* = −13→13
49734 measured reflections	*k* = −12→12
3169 independent reflections	*l* = −18→18

### 4,4'-{[Anthracene-9,10-diylbis(methylene)]bis(oxy)}dibenzoic acid dimethylformamide disolvate (2) Refinement

**Table:**

Refinement on *F*^2^	Primary atom site location: dual
Least-squares matrix: full	Secondary atom site location: difference Fourier map
*R*[*F*^2^ > 2σ(*F*^2^)] = 0.069	Hydrogen site location: inferred from neighbouring sites
*wR*(*F*^2^) = 0.182	H-atom parameters constrained
*S* = 1.02	*w* = 1/[σ^2^(*F*_o_^2^) + (0.0696*P*)^2^ + 0.9349*P*] where *P* = (*F*_o_^2^ + 2*F*_c_^2^)/3
3169 reflections	(Δ/σ)_max_ < 0.001
211 parameters	Δρ_max_ = 0.31 e Å^−^^3^
0 restraints	Δρ_min_ = −0.24 e Å^−^^3^

### 4,4'-{[Anthracene-9,10-diylbis(methylene)]bis(oxy)}dibenzoic acid dimethylformamide disolvate (2) Special details

**Table:**

Geometry. All esds (except the esd in the dihedral angle between two l.s. planes) are estimated using the full covariance matrix. The cell esds are taken into account individually in the estimation of esds in distances, angles and torsion angles; correlations between esds in cell parameters are only used when they are defined by crystal symmetry. An approximate (isotropic) treatment of cell esds is used for estimating esds involving l.s. planes.

### 4,4'-{[Anthracene-9,10-diylbis(methylene)]bis(oxy)}dibenzoic acid dimethylformamide disolvate (2) Fractional atomic coordinates and isotropic or equivalent isotropic displacement parameters (Å^2^)

**Table:**

	*x*	*y*	*z*	*U*_iso_*/*U*_eq_	
O1	0.4625 (2)	0.3352 (2)	0.09997 (14)	0.0337 (6)	
O2	0.3683 (2)	0.8009 (2)	0.38061 (15)	0.0411 (6)	
O3	0.4259 (2)	0.9259 (2)	0.26840 (16)	0.0466 (7)	
H3	0.420178	0.991287	0.304515	0.070*	
O4	0.4165 (2)	1.1349 (2)	0.37398 (17)	0.0503 (7)	
N1	0.3426 (3)	1.1942 (3)	0.50255 (19)	0.0422 (7)	
C7	0.3906 (3)	0.0820 (3)	−0.0203 (2)	0.0250 (7)	
C1	0.4710 (3)	0.0961 (3)	0.0652 (2)	0.0255 (7)	
C9	0.4409 (3)	0.4460 (3)	0.1518 (2)	0.0303 (8)	
C2	0.5807 (3)	0.0166 (3)	0.0859 (2)	0.0259 (7)	
C13	0.3834 (3)	0.5569 (3)	0.2809 (2)	0.0316 (8)	
H13	0.353304	0.551391	0.336760	0.038*	
C14	0.3968 (3)	0.4365 (3)	0.2335 (2)	0.0313 (8)	
H14	0.376246	0.349338	0.256255	0.038*	
C6	0.7205 (3)	−0.1639 (3)	0.0447 (2)	0.0312 (8)	
H6	0.740356	−0.229858	0.002326	0.037*	
C8	0.4362 (3)	0.1987 (3)	0.1318 (2)	0.0299 (7)	
H8A	0.346404	0.190190	0.134842	0.036*	
H8B	0.485761	0.181983	0.193453	0.036*	
C3	0.6662 (3)	0.0280 (3)	0.1708 (2)	0.0336 (8)	
H3A	0.649564	0.092861	0.215009	0.040*	
C15	0.4000 (3)	0.8076 (3)	0.3059 (2)	0.0368 (8)	
C12	0.4126 (3)	0.6845 (3)	0.2495 (2)	0.0309 (8)	
C10	0.4707 (3)	0.5746 (3)	0.1194 (2)	0.0335 (8)	
H10	0.501582	0.580681	0.063815	0.040*	
C11	0.4557 (3)	0.6929 (3)	0.1676 (2)	0.0354 (8)	
H11	0.474846	0.780492	0.144782	0.042*	
C5	0.7977 (3)	−0.1500 (4)	0.1261 (2)	0.0378 (8)	
H5	0.870193	−0.206227	0.140562	0.045*	
C4	0.7702 (3)	−0.0514 (4)	0.1897 (2)	0.0410 (9)	
H4	0.825451	−0.040790	0.246538	0.049*	
C16	0.3683 (3)	1.1049 (4)	0.4424 (3)	0.0443 (9)	
H16	0.349206	1.010445	0.450942	0.053*	
C17	0.3683 (4)	1.3394 (4)	0.4945 (3)	0.0541 (11)	
H17A	0.433156	1.351418	0.457255	0.081*	
H17B	0.397436	1.378518	0.555323	0.081*	
H17C	0.291892	1.387089	0.465295	0.081*	
C18	0.2832 (4)	1.1503 (4)	0.5779 (3)	0.0540 (11)	
H18A	0.198205	1.188036	0.569478	0.081*	
H18B	0.331843	1.184320	0.635586	0.081*	
H18C	0.279500	1.049111	0.579376	0.081*	

### 4,4'-{[Anthracene-9,10-diylbis(methylene)]bis(oxy)}dibenzoic acid dimethylformamide disolvate (2) Atomic displacement parameters (Å^2^)

**Table:**

	*U*^11^	*U*^22^	*U*^33^	*U*^12^	*U*^13^	*U*^23^
O1	0.0475 (14)	0.0227 (12)	0.0350 (12)	−0.0027 (10)	0.0182 (10)	−0.0034 (10)
O2	0.0483 (15)	0.0389 (14)	0.0400 (14)	−0.0043 (11)	0.0181 (12)	−0.0065 (11)
O3	0.0656 (17)	0.0302 (14)	0.0507 (15)	−0.0051 (12)	0.0288 (14)	−0.0078 (12)
O4	0.0652 (17)	0.0386 (15)	0.0510 (16)	−0.0080 (13)	0.0210 (14)	−0.0086 (12)
N1	0.0441 (18)	0.0409 (19)	0.0423 (17)	0.0027 (14)	0.0098 (14)	−0.0051 (15)
C7	0.0250 (16)	0.0212 (16)	0.0303 (17)	−0.0032 (13)	0.0094 (14)	0.0010 (13)
C1	0.0303 (17)	0.0182 (16)	0.0307 (18)	−0.0031 (13)	0.0127 (14)	0.0004 (13)
C9	0.0343 (18)	0.0233 (18)	0.0336 (18)	−0.0003 (14)	0.0067 (15)	−0.0047 (14)
C2	0.0303 (17)	0.0198 (17)	0.0288 (17)	−0.0061 (13)	0.0087 (14)	0.0007 (13)
C13	0.0363 (19)	0.0305 (19)	0.0299 (18)	−0.0025 (15)	0.0111 (15)	−0.0001 (15)
C14	0.0357 (18)	0.0251 (18)	0.0342 (19)	−0.0054 (14)	0.0092 (15)	0.0015 (14)
C6	0.0329 (18)	0.0256 (18)	0.0373 (19)	0.0005 (14)	0.0123 (15)	0.0019 (14)
C8	0.0346 (18)	0.0242 (18)	0.0318 (17)	−0.0009 (14)	0.0087 (14)	0.0019 (14)
C3	0.0365 (19)	0.034 (2)	0.0316 (19)	−0.0036 (16)	0.0103 (15)	−0.0064 (15)
C15	0.039 (2)	0.033 (2)	0.041 (2)	−0.0043 (16)	0.0153 (17)	−0.0025 (17)
C12	0.0315 (18)	0.0288 (19)	0.0338 (18)	−0.0002 (14)	0.0096 (14)	−0.0025 (15)
C10	0.041 (2)	0.033 (2)	0.0285 (18)	−0.0005 (15)	0.0106 (15)	−0.0011 (15)
C11	0.042 (2)	0.0290 (19)	0.0372 (19)	−0.0008 (15)	0.0112 (16)	0.0018 (16)
C5	0.0330 (19)	0.042 (2)	0.039 (2)	0.0063 (16)	0.0078 (16)	0.0054 (17)
C4	0.035 (2)	0.052 (2)	0.034 (2)	0.0015 (18)	0.0020 (16)	−0.0005 (17)
C16	0.042 (2)	0.040 (2)	0.052 (2)	−0.0043 (17)	0.0091 (19)	−0.0013 (19)
C17	0.075 (3)	0.039 (2)	0.052 (2)	0.004 (2)	0.019 (2)	−0.0032 (18)
C18	0.062 (3)	0.057 (3)	0.050 (2)	−0.001 (2)	0.026 (2)	0.001 (2)

### 4,4'-{[Anthracene-9,10-diylbis(methylene)]bis(oxy)}dibenzoic acid dimethylformamide disolvate (2) Geometric parameters (Å, º)

**Table:**

O1—C9	1.366 (3)	C6—C5	1.348 (4)
O1—C8	1.448 (3)	C6—H6	0.9500
O2—C15	1.225 (4)	C8—H8A	0.9900
O3—C15	1.326 (4)	C8—H8B	0.9900
O3—H3	0.8400	C3—C4	1.353 (4)
O4—C16	1.262 (4)	C3—H3A	0.9500
N1—C16	1.311 (4)	C15—C12	1.477 (4)
N1—C17	1.442 (5)	C12—C11	1.387 (4)
N1—C18	1.457 (4)	C10—C11	1.377 (4)
C7—C1	1.414 (4)	C10—H10	0.9500
C7—C6^i^	1.435 (4)	C11—H11	0.9500
C7—C2^i^	1.439 (4)	C5—C4	1.415 (5)
C1—C2	1.405 (4)	C5—H5	0.9500
C1—C8	1.499 (4)	C4—H4	0.9500
C9—C14	1.391 (4)	C16—H16	0.9500
C9—C10	1.394 (4)	C17—H17A	0.9800
C2—C3	1.431 (4)	C17—H17B	0.9800
C13—C12	1.377 (4)	C17—H17C	0.9800
C13—C14	1.383 (4)	C18—H18A	0.9800
C13—H13	0.9500	C18—H18B	0.9800
C14—H14	0.9500	C18—H18C	0.9800
			
C9—O1—C8	117.8 (2)	C2—C3—H3A	119.2
C15—O3—H3	109.5	O2—C15—O3	123.0 (3)
C16—N1—C17	121.1 (3)	O2—C15—C12	123.0 (3)
C16—N1—C18	121.0 (3)	O3—C15—C12	114.0 (3)
C17—N1—C18	117.9 (3)	C13—C12—C11	119.1 (3)
C1—C7—C6^i^	121.9 (3)	C13—C12—C15	118.6 (3)
C1—C7—C2^i^	120.0 (3)	C11—C12—C15	122.3 (3)
C6^i^—C7—C2^i^	118.1 (3)	C11—C10—C9	120.2 (3)
C2—C1—C7	120.4 (3)	C11—C10—H10	119.9
C2—C1—C8	121.6 (3)	C9—C10—H10	119.9
C7—C1—C8	118.1 (3)	C10—C11—C12	120.1 (3)
O1—C9—C14	124.4 (3)	C10—C11—H11	119.9
O1—C9—C10	115.5 (3)	C12—C11—H11	119.9
C14—C9—C10	120.1 (3)	C6—C5—C4	119.8 (3)
C1—C2—C3	122.7 (3)	C6—C5—H5	120.1
C1—C2—C7^i^	119.6 (3)	C4—C5—H5	120.1
C3—C2—C7^i^	117.7 (3)	C3—C4—C5	120.9 (3)
C12—C13—C14	122.0 (3)	C3—C4—H4	119.5
C12—C13—H13	119.0	C5—C4—H4	119.5
C14—C13—H13	119.0	O4—C16—N1	124.9 (3)
C13—C14—C9	118.4 (3)	O4—C16—H16	117.6
C13—C14—H14	120.8	N1—C16—H16	117.6
C9—C14—H14	120.8	N1—C17—H17A	109.5
C5—C6—C7^i^	122.0 (3)	N1—C17—H17B	109.5
C5—C6—H6	119.0	H17A—C17—H17B	109.5
C7^i^—C6—H6	119.0	N1—C17—H17C	109.5
O1—C8—C1	107.4 (2)	H17A—C17—H17C	109.5
O1—C8—H8A	110.2	H17B—C17—H17C	109.5
C1—C8—H8A	110.2	N1—C18—H18A	109.5
O1—C8—H8B	110.2	N1—C18—H18B	109.5
C1—C8—H8B	110.2	H18A—C18—H18B	109.5
H8A—C8—H8B	108.5	N1—C18—H18C	109.5
C4—C3—C2	121.6 (3)	H18A—C18—H18C	109.5
C4—C3—H3A	119.2	H18B—C18—H18C	109.5
			
C6^i^—C7—C1—C2	−178.4 (3)	C7^i^—C2—C3—C4	−0.5 (4)
C2^i^—C7—C1—C2	1.5 (4)	C14—C13—C12—C11	0.5 (5)
C6^i^—C7—C1—C8	1.0 (4)	C14—C13—C12—C15	−177.8 (3)
C2^i^—C7—C1—C8	−179.1 (2)	O2—C15—C12—C13	2.2 (5)
C8—O1—C9—C14	0.8 (4)	O3—C15—C12—C13	−177.7 (3)
C8—O1—C9—C10	178.4 (3)	O2—C15—C12—C11	−176.1 (3)
C7—C1—C2—C3	179.2 (3)	O3—C15—C12—C11	4.0 (5)
C8—C1—C2—C3	−0.2 (4)	O1—C9—C10—C11	−178.3 (3)
C7—C1—C2—C7^i^	−1.5 (4)	C14—C9—C10—C11	−0.6 (5)
C8—C1—C2—C7^i^	179.2 (3)	C9—C10—C11—C12	1.0 (5)
C12—C13—C14—C9	0.0 (5)	C13—C12—C11—C10	−1.0 (5)
O1—C9—C14—C13	177.6 (3)	C15—C12—C11—C10	177.3 (3)
C10—C9—C14—C13	0.1 (5)	C7^i^—C6—C5—C4	−0.6 (5)
C9—O1—C8—C1	−178.3 (2)	C2—C3—C4—C5	−0.6 (5)
C2—C1—C8—O1	104.5 (3)	C6—C5—C4—C3	1.1 (5)
C7—C1—C8—O1	−74.8 (3)	C17—N1—C16—O4	0.2 (5)
C1—C2—C3—C4	178.9 (3)	C18—N1—C16—O4	178.0 (3)

Symmetry code: (i) −*x*+1, −*y*, −*z*.

### 4,4'-{[Anthracene-9,10-diylbis(methylene)]bis(oxy)}dibenzoic acid dimethylformamide disolvate (2) Hydrogen-bond geometry (Å, º)

*Cg*2 is the centroid of the C9–C14 ring of (2).

**Table:**

*D*—H···*A*	*D*—H	H···*A*	*D*···*A*	*D*—H···*A*
O3—H3···O4	0.84	1.74	2.575 (3)	175
C8—H8*A*···O2^ii^	0.99	2.54	3.429 (4)	149
C16—H16···O2	0.95	2.31	3.080 (4)	138
C4—H4···*Cg*2	0.95	3.07	3.96	157

Symmetry code: (ii) −*x*+1/2, *y*−1/2, −*z*+1/2.
